# Incidence and percentage of survival after cardiac arrest outside and inside hospital: A comparison between two regions in Sweden

**DOI:** 10.1016/j.resplu.2024.100594

**Published:** 2024-03-06

**Authors:** A. Strömsöe, J. Herlitz

**Affiliations:** a School of Health and Welfare, Dalarna University, S-79188 Falun, Sweden; b Center for Clinical Research Dalarna, Uppsala University, S-79182 Falun, Sweden; c Department of Prehospital Care, Region of Dalarna, S-79129 Falun, Sweden; d Department of Caring Science, University of Borås, S-50190 Borås, Sweden

**Keywords:** Out-of-hospital cardiac arrest, In-hospital cardiac arrest, Incidence, Resuscitation, Survivors

## Abstract

**Aim:**

To compare the incidence and percentage of survival after cardiac arrest outside and inside hospital where cardiopulmonary resuscitation (CPR) had been started between two regions in Sweden in a 10-year perspective.

**Methods:**

A retrospective observational study including CPR treated patients both after out-of-hospital and in-hospital cardiac arrest (OHCA and IHCA) in Sweden, 2013–2022. Data was retrieved from the Swedish Registry of Cardiopulmonary Resuscitation (SRCR).

**Results:**

The overall incidence of OHCA and IHCA events were 2,940 in Dalarna (having a lower population and population density) and 16,187 in Västra Götaland (having a higher population and population density). The overall incidence of survival when OHCA and IHCA were combined was 20 per 100,000 person years in Dalarna and 19 per 100,000 person years in Västra Götaland. The corresponding result for OHCA was 9 versus 7 and for IHCA 11 versus 12. The overall percentage of survival was 20% in Dalarna and 19% in Västra Götaland. The corresponding result for OHCA was 13% versus 10% and for IHCA 37% versus 36%.

**Conclusion:**

Overall, there was no marked difference neither in incidence nor in percentage of survival after cardiac arrest between the two regions. However, regarding cardiac arrest that took place outside hospital both incidence and percentage of survival was higher in Dalarna than in Västra Götaland despite the fact that the former had lower population density.

## Introduction

Sudden death is one of the most frequent mechanisms of death in a global perspective. Sudden cardiac arrest is usually divided into out-of-hospital cardiac arrest (OHCA) and in-hospital cardiac arrest (IHCA). The prognosis between patients who suffer from OHCA and those who suffer from IHCA has been reported to differ with a higher survival rate after IHCA.^1^

Not all patients who suffer from a sudden cardiac arrest are exposed to cardiopulmonary resuscitation (CPR).^2^ There are a number of reasons why this is not the case. Among patients with OHCA the most common reason for not starting CPR after cardiac arrest is probably that the event is non-witnessed and therefore the delay time until CPR can be started is too long for a meaningful resuscitation.

Among patients with IHCA the most common reason why CPR is not started is most likely a Do Not Attempt Resuscitation (DNAR) order, which means that a decision has already been made not to start CPR in case of cardiac arrest due frailty and/or severe comorbidity or a very high age making CPR unethical and less meaningful.^3^

Due to the reasons given above the survival after a sudden cardiac arrest is mostly related to the number of patients in whom CPR was started and is expressed as percentage. However, the denominator may vary based on a number of factors, including local routines, logistics and cultural factors, and therefore another way of calculating the survival after a sudden cardiac arrest is to estimate the incidence of survival per 100,000 person years.^4^, ^5^, ^6^, ^7^

In the majority of reports dealing with survival after sudden cardiac arrest results are presented separately for OHCA and for IHCA. But the education of CPR in a community will most often include health care providers as well as lay persons.^8^, ^9^ Therefore, in this study we aimed at reporting the incidence and percentage of survival from OHCA and IHCA added together. With such an approach the overall success of CPR within a community can be estimated and reported back to the community.

Thus, the primary aim of the present study was comparing the incidence and percentage of survival after cardiac arrest where CPR had been started regardless of whether it took place outside or inside hospital in a 10-year perspective between two regions in Sweden reflecting different geography and different population density. But results will also be presented for OHCA and IHCA separately.

## Methods

### Design and setting

A retrospective observational study was performed with a quantitative approach.

In Sweden there are approximately 10 million inhabitants who live in 21 different regions. We included all patients suffering from cardiac arrest in the two regions, Dalarna and Västra Götaland which were reported to the Swedish Registry of Cardiopulmonary Resuscitation (SRCR) during 2013-2022.^10^ The population in Sweden has increased from 9, 596 436 (2013) to 10, 545 310 (2022).^11^ In Dalarna the population has increased from 277,118 (2013) to 288,343 (2022) and in Västra Götaland the population has increased from 1,613342 (2013) to 1,757225 (2022). The two regions represented more sparsely areas in Dalarna versus more urban areas in Västra Götaland. Dalarna is localized in the lower part of Northern Sweden and has a population density of 10.3 per square kilometer and an area of 28,030 square kilometers.

Västra Götaland is localized in the western part of the southern Sweden and has a population density of 73.9 per square kilometer and an area of 23,800 square kilometer.^11^ The data has been reported by the emergency medical service (EMS) crew (OHCA) and the IHCA cases have been reported by the Health Care Providers (HCP). The two regions were selected in order to compare a region with mostly sparsely populated areas with a region with more urban areas.

### The Swedish Registry of Cardiopulmonary Resuscitation (SRCR)

The SRCR was initiated 1990 with the OHCA part and later with the IHCA part (2006). The SRCR is classified as a national quality registry with a financial support from the Swedish government and the Swedish Association of Local Authorities and Regions. The SRCRś main purpose is to provide data for the development of the Swedish health care in terms of assessment and treatment of cardiac arrest. The registry also serves as feedback to the popular movement of CPR which has been growing since 1983. Over time, the reported data has increased, and all the EMS systems and hospitals report to SRCR. Initially, the most common way to report data was as a percentage, regardless of completeness of the data. The reporting of OHCA takes place digitally while the IHCA cases are reported both in paper form and digitally.

### Data collection

The inclusion criteria for being reported to the SRCR is cardiac arrest where CPR was attempted. The formal definition of a cardiac arrest is “Unconsciousness combined with absence of or abnormal respiration”. But in reality, is the inclusion of patients based on whether CPR was started or not as highlighted by the name of the registry. The registry is divided into two parts, 1) patients with OHCA and 2) patients with IHCA. Both parts were included in the study.

All variables that were reported, were reported in total (OHCA and IHCA were added when possible) and separately for OHCA and IHCA when possible.

The primary endpoints were the incidence of survival to 30 days per 100,000 person years and percentage of survival when OHCA and IHCA were added. Secondary endpoints were incidence of survival and percentage of survival for OHCA and for IHCA respectively. Incidence of survival was defined as the number of survivors per 100, 000 person years. Percentage of survival was defined as the number of survivors divided by the number or resuscitation attempts as reported to the registry.

We also reported the number of events per 100,000 person years for OHCA and for IHCA respectively. Victims in the two regions were compared in total and separately for OHCA and IHCA regarding some further variables. They were: 1) age,2) sex, 3) place (defined as at home for OHCA (yes/no) and in monitored wards for IHCA (yes/no), 4) witnessed (yes/no) both for OHCA and IHCA including both bystander witnessed and crew witnessed for OHCA, 5) initial rhythm - ventricular fibrillation/tachycardia, VF/VT (yes/no) both for OHCA and IHCA.

The following time intervals were compared between the two regions: 1) collapse-call for OHCA and for IHCA, 2) collapse - start of CPR for OHCA and for IHCA, 3) collapse - defibrillation among patients found in VF/VT for OHCA and for IHCA and 4) dispatch of EMS - arrival of EMS at patients’ side only for OHCA. Finally, we described the change over time from 2013 until 2022 in the two regions with regard to percentage of survival both for the combination of OHCA and IHCA as well as separately for the two.

### Statistical analysis

Descriptive statistics were used in this study as percentage, median, mean, quartiles and standard deviation (SD). Furthermore, data was presented as incidence per 100,000 person years (OHCA and IHCA). Nonparametric statistical methods were used as Chi-squared test for categorial variables and for continuous variables, the Mann-Whitney U-test was used. A p-value of less than 0.05 was regarded as significant and a two-tailed test was applied.

### Ethical considerations

Ethical permission for the study was authorized by the Swedish Ethical Review Authority (Dnr 2023–05520-01).

## Results

In all, there were 2,940 events in Dalarna and 16,187 events in Västra Götaland between 2013 and 2022. The corresponding results for OHCA were 2,059 respective 10,682 and for IHCA 881 respective 5,505.

Baseline characteristics (Table 1).

**Table 1:** Baseline characteristics of treated out-of-hospital and in-hospitals cardiac arrest in Sweden, 2013–2022.

**TABLE 1. BASELINE CHARACTERISTICS**									
	**ALL**			**OHCA**			**IHCA**		
	**Dalarna**	**Västra Götaland**	**p-value**	**Dalarna**	**Västra Götaland**	**p-value**	**Dalarna**	**Västra Götaland**	**p-value**
Number of events (n)	2,940	16,187		2,059	10,682		881	5,505	
Incidence per 100,000 person years	104	97		73	64		31	33	
Age years Median/mean/SD)	73/70/16.5	73/69/19.9	0.597	72/69/17.4	72/68/18.2	0.856	74/72/14	74/70/17	0.315
Male (n/%)	1,888/64	10,368/64	0.895	1,358/66	6,916/65	0.317	530/60	3,452/63	0.147
Witnessed (n/%)	1,946/67	11,117/70	0.002	1,234/60	6,630/62	0.029	712/81	5,443/82	0.444
Initial rhythm VT/VF (n/%)	851/29	4,638/29	0.720	380/21	2,040/21	0.776	471/53	2,598/47	0.405
Place Home (n/%) Monitored wards (n/%)				1,428/69	7,912/74	<0.001	548/62	2,871/52	<0.001

The overall incidence of cardiac arrest was 104 per 100, 000 person years in Dalarna and 97 in Västra Götaland. The corresponding values for OHCA were 73 versus 64 and for IHCA 31 versus 33. Neither age, sex, initial rhythm nor witnessed events differed markedly between the two regions. In terms of place, a higher proportion of OHCA events took place in patients’ home in Västra Götaland. Conversely, the proportion of IHCA events that took place in monitored wards was higher in Dalarna.

Delay times (Table 2).

**Table 2:** Delay times and treatment of treated out-of-hospital and in-hospitals cardiac arrest in Sweden, 2013–2022.

**TABLE 2. DELAY TIMES AND TREATMENT**									
	**ALL**			**OHCA**			**IHCA**		
	**Dalarna**	**Västra Götaland**	**p-value**	**Dalarna**	**Västra Götaland**	**p-value**	**Dalarna**	**Västra Götaland**	**p-value**
Collapse-Call*, ** Median Q1-Q3, Mean (SD)	1 0–2 1.96 (3.3)	1 0–2 2.23 (4.31)	0.197	2 1–4 3.12 (3.98)	2 1–4 3.31 (5.13)	0.264	0 0–1 1.11 (2.69)	0 0–1 0.91 (2.36)	0.542
Collapse-CPR Median Q1-Q3 Mean (SD)	1 0–4 3.6 (6.6)	1 0–5 4 (7.22)	0.006	2 0–8 5.18 (7.54)	2 0–9 5.75 (8.25)	0.067	0 0–1 0.38 (0.95)	0 0–1 0.58 (1.84)	<0.001
Collapse-Defibrillation Median Q1-Q3 Mean (SD)	10 2–18 12.1 (9.36)	10 2–19 12.23 (11.89)	0.937	15 9–23 16.88 (11.97)	15 9–23 16.81 (11.75)	0.997	2 1–4 3.85 (5.97)	2 1–4 3.68 (5.90)	0.126
EMS Response Time Dispatch-EMS arrival*** Median Q1-Q3 Mean (SD)				11 7–18 13.59 (8.75)	11 7–15 12.23 (7.23)	<0.001			

The time from collapse until call for an ambulance in OHCA did not differ between the two regions. Furthermore, the corresponding values for IHCA and for the overall did not differ between the two regions. The time between collapse until start of CPR differed slightly for the two regions where Dalarna had a faster start of CPR. The time from collapse until defibrillation did not differ whereas the EMS response time was slightly shorter in Dalarna.

Outcome (Table 3).

**Table 3:** Outcome of treated out-of-hospital and in-hospitals cardiac arrest in Sweden, 2013–2022.

**TABLE 3. OUTCOME**						
	**ALL**		**OHCA**		**IHCA**	
	**Dalarna**	**Västra Götaland**	**Dalarna**	**Västra Götaland**	**Dalarna**	**Västra Götaland**
Survival 30 days (n/%)	587/20	3,126/19	265/13	1,120/10	322/37	2,006/36
Survivors (per 100,000 person years)	20	19	9	7	11	12

The overall incidence of survival to 30 days was 20 per 100,000 person years in Dalarna versus 19 in Västra Götaland. The corresponding result for OHCA was 9 versus 7 respective for IHCA 11 versus 12. In terms of percentage survival for 30 days there was no difference when OHCA and IHCA results were combined, but among patients with OHCA the survival rate was higher in Dalarna (13% versus 10%).

Change over time (Table 4).

**Table 4:** Changes over time in survival and percentage in Dalarna and Västra Götaland.

**TABLE 4. CHANGES OVER TIME IN SURVIVAL - PERCENTAGE IN DALARNA AND VÄSTRA GÖTALAND**											
**Year**	**2013**	**2014**	**2015**	**2016**	**2017**	**2018**	**2019**	**2020**	**2021**	**2022**	**p-value**
**All**											
Dalarna	22	24	17	21	18	19	21	22	18	21	0.253
Västra Götaland	19	21	18	19	18	19	20	18	19	22	
**OHCA**											
Dalarna	15	14	9	13	14	10	14	13	14	14	0.011
Västra Götaland	11	11	10	10	11	10	11	9	11	12	
**IHCA**											
Dalarna	36	42	26	40	29	39	40	41	29	39	0.481
Västra Götaland	34	40	32	34	34	37	38	37	38	39	

Survival did not change over time, neither among OHCA nor IHCA cases.

## Discussion

The major aim of this study was to report on the incidence and percentage of survival to 30 days after the combined analysis of cardiac arrest outside and inside hospital in two regions in Sweden with a different population density.

Overall, there was no marked difference in the percentage of survival between the two regions. When it comes to the incidence of survival, the result showed a difference in numbers of survivors between the two regions, in the overall, the OHCA and the IHCA. In terms of OHCA the incidence as well as the percentage of survival was higher in Dalarna despite the lower population density in this region. A previous study showed no significant association between population density and survival after OHCA in a national perspective in Sweden.^12^

The incidence of survival is dependent both on the incidence of cardiac arrest events i.e the reported number of events as well as the percentage of survival i.e the proportion of survivors among all patients where resuscitation was attempted. Both of these two estimates of survival were higher in Dalarna than in Västra Götaland after OHCA. One may speculate that the prevalence of cardiovascular disease may differ between regions in Sweden and thereby influence incidence of cardiac arrest events. Such a difference has been suggested with a higher prevalence of cardiovascular disease in the northern Sweden.^13^ Mechanisms behind such differences may include presence of risk factors, climate, lifestyle and genetics.

We have no clear explanation why the percentage of survival was higher in Dalarna after OHCA. A number of factors may influence the percentage of survival including the patient’s comorbidity^14^ and frailty^15^ as well as the quality of CPR that was given.

In terms of IHCA, the percentage of survival appears to be similar in the two regions but differs when it comes to the incidence of survival and Västra Götaland seems to have a slightly higher number of survivors. As expected, was the incidence of survival and particularly the percentage of survival much higher after IHCA than after OHCA in both regions which is in agreement with previous reports.^1^

An important result in this survey is the overall incidence of survival when OHCA and IHCA are combined. This finding gives an implication on how many lives that overall could be saved in a community after resuscitation and could therefore be looked upon as the overall consequence of CPR training in a community including lay persons as well as health care providers. To the best of our knowledge such a consequence of the CPR movement has never been reported before in the CPR literature.

The proportion of patients having an initial rhythm of VT/VF after IHCA was higher in Dalarna than in Västra Götaland. However, the results show that the location of OHCA is more common in residential areas in Västra Götaland, which is an overall challenge when it comes to influencing the chance of survival.^16^

### Clinical implications

With the finding of a higher incidence as well as percentage of survival after cardiac arrest outside hospital in a region with a sparser population density, it might be of importance to focus on the factors that are most important for an increased chance of survival. A higher proportion of witnessed cases and reduced delay times from call to initiation of CPR including defibrillation are examples of factors that will influence the chance of achieving a higher survival rate. However, neither of these two factors differed markedly between the two regions. But the message is clear, with a good organization many lives can be saved after OHCA even in a region with a low population density.

### Limitations

There is a lack of reporting particularly of IHCA cases to the SRCR which has led to internal missing that might affect the quality of the data. The statistical analyses in this study will not consider the missing cases. The major reason for lack of reporting cases of IHCA is that the rescue team is not always called upon after a cardiac arrest, particularly in the intensive care unit and then reporting to the registry may be forgotten. The overall percentage of cases with IHCA where CPR was attempted which are not reported to the registry has been estimated to amount to about 20%.

## Conclusion

When comparing the incidence and the percentage of survival after cardiac arrest outside and inside hospital between two regions in Sweden with a different population density there was no major difference overall. However, surprisingly there was a higher incidence and percentage of survival in the region with a lower population density. The mechanisms behind this finding need to be further explored.

## Conflicts of interest

None. J.H. is the deputy registrar of the SRCR. A.S. is the coordinator of the SRCR.

## CRediT authorship contribution statement

**Anneli Strömsöe:** Writing – review and editing, Writing – original draft, Visualization, Validation, Project administration, Methodology, Formal analysis, Data curation, Conceptualization. **Johan Herlitz:** Writing – review and editing, Resources, Methodology, Formal analysis, Data curation, Conceptualization.

## Declaration of competing interest

The authors declare that they have no known competing financial interests or personal relationships that could have appeared to influence the work reported in this paper.
